# Cognitive impairments in first-episode psychosis patients with attenuated niacin response

**DOI:** 10.1016/j.scog.2025.100346

**Published:** 2025-01-27

**Authors:** MingLiang Ju, Bin Long, YanYan Wei, XiaoChen Tang, LiHua Xu, RanPiao Gan, HuiRu Cui, YingYing Tang, ZhengHui Yi, HaiChun Liu, ZiXuan Wang, Tao Chen, Jin Gao, Qiang Hu, LingYun Zeng, ChunBo Li, JiJun Wang, HuanZhong Liu, TianHong Zhang

**Affiliations:** a Department of Psychiatry, Chaohu Hospital of Anhui Medical University, Hefei 238000, China; b Shanghai Mental Health Center, Shanghai Jiaotong University School of Medicine, Shanghai Engineering Research Center of Intelligent Psychological Evaluation and Intervention, Shanghai Key Laboratory of Psychotic Disorders, Shanghai 200030, China; c Department of Automation, Shanghai Jiao Tong University, Shanghai 200240, China; d Shanghai Xinlianxin Psychological Counseling Center, Shanghai, China; e Big Data Research Lab, University of Waterloo, Ontario, Canada; f Labor and Worklife Program, Harvard University, Cambridge, MA, United States; g Department of Clinical Psychology, Qilu Hospital (Qingdao), Cheeloo College of Medicine, Shandong University, Qingdao, Shandong, China; h Department of Psychiatry, ZhenJiang Mental Health Center, Zhenjiang, China; i Department of Psychiatric Rehabilitation, Shenzhen Kangning Hospital, ShenZhen, GuangDong, China; j Department of Psychiatry, Nantong Fourth People's Hospital & Nantong Brain Hospital, Suzhou 226000, China

**Keywords:** Psychosis, Neurocognition, Niacin skin test, Endophenotype, Biotype

## Abstract

**Background:**

Psychosis is a complex brain disorder with diverse biological subtypes influenced by various pathogenic mechanisms, which can affect treatment efficacy. The ANR(Attenuated Niacin Response) subtype is characterized by pronounced negative symptoms and functional impairments, suggesting a distinct clinical profile. However, research on the cognitive characteristics associated with the ANR subtype in drug-naïve first-episode psychosis(FEP) patients remains limited.

**Methods:**

This observational study involved 54 FEP patients and 52 healthy controls(HC). Clinical psychopathology was assessed using the Positive and Negative Syndrome Scale(PANSS), while cognitive performance was evaluated through the Chinese version of the MATRICS Consensus Cognitive Battery(MCCB). Additionally, niacin response was measured using aqueous methylnicotinate patches, with responses quantified to classify participants into ANR or normal niacin response (NNR) groups.

**Results:**

Among the FEP patients, 25.9 % were classified as having ANR, significantly higher than the 7.7 % in the HC group (*χ*^*2*^ = 6.247, *p* = 0.012). The ANR group exhibited more severe negative symptoms and higher total PANSS scores compared to the NNR group, with significant differences in cognitive performance on the Trail Making test and the Brief Visuospatial Memory Test-Revised. Correlation analyses revealed a significant positive relationship between overall symptom severity and niacin response, as well as between cognitive performance and niacin response, particularly for the Trail Making and Symbol coding tests.

**Conclusions:**

This study demonstrates that the ANR subtype in first-episode psychosis is linked to more severe negative symptoms and cognitive impairments. Targeted assessments and interventions for patients with ANR may improve treatment outcomes and enhance understanding of cognitive dysfunction in psychotic disorders.

## 1 Introduction

Psychosis is a complex brain disorder, and its pathogenesis remains largely based on various unverified hypotheses. Different pathogenic mechanisms lead to the existence of distinct biological subtypes within the diagnostic category of psychotic disorders (Boerrigter et al., 2017; Clementz et al., 2022). Even when the same treatment is applied, these subtypes may exhibit varying degrees of efficacy (Clementz et al., 2023; Clementz et al., 2020). Niacin skin response blunting serves as an internal phenotypic biomarker for psychotic disorders, demonstrating a close association with the condition, a higher prevalence among first-degree relatives, and heritability, making it a potential trait marker (Hu et al., 2022; Messamore, 2018). The mechanism underlying niacin skin response blunting shares a common biological basis with the pathogenesis of schizophrenia, specifically involving abnormalities in phospholipid metabolism (Yang et al., 2021). A substantial body of evidence suggests the presence of a biological subtype in schizophrenia patients, whose underlying mechanisms may be related to inflammation and oxidative stress (Ansarey, 2021; Zhang et al., 2023c), and these patients may exhibit attenuated niacin response (ANR). These processes are known to disrupt the normal functioning of neural circuits, which are essential for cognitive processes such as attention, memory, and executive function. As a result, in FEP patients with ANR, the heightened levels of inflammation and oxidative stress might contribute significantly to the observed cognitive impairments, highlighting a potential link between ANR and cognitive dysfunction.

Patients with the ANR subtype of psychotic disorders exhibit greater clinical homogeneity, characterized primarily by more pronounced negative symptoms (such as affective flattening and poverty of thought) (Zhang et al., 2023a) and functional impairments (Messamore, 2012). Smesny et al. (Smesny et al., 2003) found that, in early psychotic disorder patients, both methods of assessing niacin skin response revealed that those with the blunted subtype exhibited significantly more negative symptoms compared to non-blunted patients. Research conducted in China with 240 first-episode psychosis (FEP) patients also indicated that individuals with the blunted subtype presented with more severe negative symptoms (Zhang et al., 2023a). Additionally, a study by Nilsson et al. (Nilsson et al., 2015) demonstrated that patients with the ANR subtype had lower cognitive function and IQ levels compared to their non-blunted counterparts. Messamore (Messamore, 2012), in an assessment of niacin skin response in stable schizophrenia patients, found that the degree of skin response blunting was associated with greater functional impairments as evaluated by blinded assessors. These findings suggest that patients with the blunted subtype of psychotic disorders may exhibit more pronounced clinical manifestations, indicating that clinicians could implement more targeted assessments and interventions based on the specific symptom clusters presented.

Research on the relationship between niacin response levels and cognitive function in FEP has been very limited, with only a few studies focusing on stabilized schizophrenia patients on long-term medication and featuring small sample sizes without control groups. These limitations have resulted in a lack of evidence regarding the cognitive characteristics of the ANR subtype in psychosis. Our previous studies indicated a connection between negative symptoms and the ANR subtype. Therefore, this research aims to conduct a comprehensive cognitive assessment of drug-naïve FEP patients, using matched healthy controls (HC) to investigate the cognitive characteristics associated with the ANR subtype in FEP.

## 2 Methods

### 2.1 Participants

This observational study included 54 patients diagnosed with FEP and 52 HC. Participants were recruited from the Shanghai Mental Health Center (SMHC) in China between 2017 and 2023. Ethical approval for the study was granted by the research ethics committee at SMHC (IRB2016–009), and all participants provided informed consent. All procedures conducted in this study complied with the ethical standards of the relevant national and institutional committees on human experimentation and the 1975 Declaration of Helsinki, revised in 2008.

Inclusion criteria for the study were as follows: (i) participants aged 18 to 45 years; (ii) no restrictions on gender; (iii) ability to provide informed consent; (iv) completion of at least six years of primary education and proficiency in Mandarin; (v) meeting DSM-IV criteria for psychotic disorders, confirmed through the Structured Clinical Interview for DSM Disorders (SCID) interview, with their first psychotic episode occurring within the past year; and (vi) being psychotropically naïve.

Exclusion criteria included: (i) the presence of serious somatic conditions, such as pneumonia, cancer, or heart failure; (ii) intellectual disabilities, namely an intelligence quotient (IQ) score below 70 on standardized tests like the Wechsler Adult Intelligence Scale; (iii) history of substance abuse or dependence (e.g., methamphetamine); (iv) infections or inflammatory conditions within the past month; (v) past or current immune system disorders, such as autoimmune diseases (e.g., systemic lupus erythematosus, rheumatoid arthritis, multiple sclerosis), immunodeficiency disorders (e.g., acquired immunodeficiency syndrome, severe combined immunodeficiency), and severe allergic disorders (e.g., anaphylaxis, chronic urticaria with severe symptoms that significantly affect immune system function); and (vi) severe allergies to niacin.

The research procedures were conducted independently of the standard clinical treatment protocols at SMHC. The 52 HC participants were recruited from the local community in Shanghai and were matched to the FEP group based on age, gender, and educational background, ensuring a comparable sample for the study.

### 2.2 Clinical and cognitive measurement

Clinical psychopathology was evaluated using the PANSS (Kay et al., 1989), comprising 30 items categorized into three subscales: positive symptoms (PANSS-P; items P1–7), negative symptoms (PANSS-N; items N1–7), and general psychopathology (PANSS-G; items G1–16). Each item was assessed on a 7-point Likert scale, where 1 indicates absence of the symptom and 7 indicates extreme severity. Structured clinical interviews were carried out by a senior psychiatrist who had completed the necessary training for this type of assessment. To make the three PANSS subscales comparable, we conducted a full sample-based *Z*-score transformation.

Neurocognitive performance was evaluated using the Chinese version of the MATRICS Consensus Cognitive Battery (MCCB) (Kern et al., 2008; Nuechterlein et al., 2008; Shi et al., 2013). The MCCB is the most widely used tool for assessing cognitive function in individuals with psychosis, including those with Clinical High Risk (CHR) (Zhang et al., 2016a; Zhang et al., 2022a; Zhang et al., 2024b) and FEP patients (Zhang et al., 2023b; Zhang et al., 2024c) in China (Zhang et al., 2024a). In line with the original MCCB, the study included eight subtests: (1) Part A of the Trail Making Test (Trail Making A); (2) symbol coding from the Brief Assessment of Cognition in Schizophrenia (BACS symbol coding); (3) Category Fluency Test; (4) Continuous Performance Test-Identical Pairs (CPT-IP); (5) spatial span from the Wechsler Memory Scale-III (WMS-3 spatial span); (6) Revised Hopkins Verbal Learning Test (HVLT-R); (7) Revised Brief Visuospatial Memory Test (BVMT-R); and (8) Mazes from the Neuropsychological Assessment Battery (NAB mazes). Due to differences in linguistic and cultural familiarity, the Maryland Letter-Number Span Task is not part of the Chinese MCCB. It is noteworthy that for all tests, higher scores indicate better cognitive functioning, except for the Trail Making A test, where lower scores suggest better cognitive performance. In this study, the social cognition domain of the MCCB was not included in the assessment. This decision was made because, in practical application for evaluating FEP patients, particularly those from the Chinese population, the social cognition component of the MCCB is not entirely appropriate. Cognitive assessments for all participants were conducted by a rater who underwent rigorous training in the MCCB. The assessment process was consistent with the guidelines outlined in the MCCB operation manual.

### 2.3 Measurement of the niacin response

A round filter paper patch was utilized to administer niacin in the form of aqueous methylnicotinate (AMN). Solutions of AMN at concentrations of 0.1 M, 0.01 M, 0.001 M, and 0.0001 M were prepared on the day of testing. To ensure a consistent reference distance, a sticky ruler was affixed to the inner side of each participant's forearm. Four wet paper patches, each containing one of the AMN solutions, were applied to adjacent sites on the forearm skin for one minute before being removed. The skin flush response was documented from a fixed vertical perspective at 5, 10, 15, and 20 min after patch removal (Sun et al., 2018). The skin flush response was assessed using the following scale: 0 for no erythema; 1 for incomplete erythema; 2 for complete erythema within the patch area; and 3 for erythema accompanied by edema beyond the defined area of the patch (Ward et al., 1998). A research assistant rated the flush responses of each participant, and these ratings were subsequently verified by a senior researcher. Both groups were blinded to the participants' grouping information. The assessment utilized a four-point scale across 16 scoring sites, with the scores from each site summed to obtain a total score. An ANR was defined as a total score of <20 and a score of <2.5 at the 15-min time point for the 10^−1^ mol/L concentration (Sun et al., 2018). Participants who did not meet the criteria for ANR were classified into the normal niacin response (NNR) group.

### 2.4 Statistical analysis

Demographic, clinical, and cognitive characteristics are presented for both the FEP and HC groups. Quantitative variables are reported as mean ± standard deviation (S.D.), while qualitative variables are shown as frequencies (%). The two groups were compared using *χ*^*2*^ tests for categorical variables and independent *t*-tests for individual PANSS item scores and cognitive variables. To provide a clearer representation of the results, clinical and cognitive variables were transformed into *Z*-scores for the entire sample and depicted using radar charts.

The niacin response level is defined as the total score derived from the 16 raw scores obtained at different AMN concentrations (0.0001, 0.001, 0.01, and 0.1 M) and at various time points (5, 10, 15, and 20 min). Participants were further categorized into ANR and NNR groups based on the niacin tests. The analysis of the correlation between niacin response levels and clinical symptoms as well as cognitive functions included the presentation of slope, F-value, and *p*-value. The slope is used to illustrate the strength and direction of the correlation, indicating how changes in niacin response are associated with variations in clinical and cognitive measures.

## 3 Results

### 3.1 Demographic and clinical characteristics

Table 1 presents the FEP group had a significantly lower mean education level (11.94 years) compared to the HC group (16.15 years, *p* < 0.001). Similarly, fathers of FEP participants had lower education levels (10.22 years) than those in the HC group (11.93 years, *p* = 0.032). Cognitive assessments revealed notable deficits in the FEP group across various measures. The FEP group scored significantly lower on the Trail Making Test A (mean: 41.85 vs. 28.48, *p* < 0.001), BACS symbol coding (46.59 vs. 67.56, *p* < 0.001), and the HVLT-R (18.94 vs. 28.06, *p* < 0.001). Other cognitive tasks, such as the WMS-3 spatial span, NAB mazes, BVMT-R, Category Fluency, and CPT-IP, also demonstrated significantly poorer performance in the FEP group (all *p*-values <0.001).

**Table 1:** Demographic, clinical and cognitive characteristics and comparisons between FEP and HC groups.

Variables	FEP		HC		Comparisons	
					*t**/χ***^***2***^	*p*
Cases [n, %]	54		52		–	–
Age (years) [mean, SD.]	24.70	8.632	27.25	9.165	−1.473	0.144
Male [n, %]	27	50.0 %	26	50.0 %	0	1
Female [n, %]	27	50.0 %	26	50.0 %		
Education (years) [mean, SD.]	11.94	3.253	16.15	5.665	−4.713	0.000
Father Education (years) [mean, SD.]	10.22	3.895	11.93	3.757	−2.177	0.032
Mother Education (years) [mean, SD.]	9.20	4.069	10.49	4.015	−1.563	0.121
Height (meter) [mean, SD.]	1.6790	0.073	1.670	0.073	0.644	0.521
Weight (kg) [mean, SD.]	59.46	10.094	61.27	9.734	−0.939	0.350
BMI [mean, SD.]	21.089	3.284	21.905	2.650	−1.404	0.163
Positive symptoms [mean, SD.]	22.94	5.496	–	–	–	–
Negative symptoms [mean, SD.]	19.59	8.747	–	–	–	–
General symptoms [mean, SD.]	44.28	6.820	–	–	–	–
PANSS total score [mean, SD.]	86.81	14.978	–	–	–	–
Trail Making A	41.85	17.023	28.48	11.020	4.781	0.000
BACS symbol coding	46.59	11.452	67.56	8.484	−10.677	0.000
HVLT-R	18.94	6.485	28.06	3.489	−8.961	0.000
WMS-3 spatial span	14.26	2.830	17.69	2.948	−6.118	0.000
NAB mazes	12.30	6.592	21.04	5.049	−7.644	0.000
BVMT-R	21.17	8.165	29.79	3.902	−6.893	0.000
Category Fluency	16.91	5.285	25.87	5.441	−8.598	0.000
CPT-IP	2.06	0.820	3.19	0.494	−8.609	0.000

Note: BMI: Body Mass Index; SD: standard deviation; BACS, Brief Assessment of Cognition in Schizophrenia symbol coding; BVMT-R, Brief Visuospatial Memory Test–Revised; CPT-IP, Continuous Performance Test–Identical Pairs; HVLT-R, Hopkins Verbal Learning Test–Revised; NAB, Neuropsychological Assessment Battery mazes; WMS-3, Wechsler Memory Scale–Third Edition spatial span.

### 3.2 Comparisons between ANR and NNR

Among the 54 FEP patients, 14 (25.9 %) were classified as having ANR, compared to 4 (7.7 %) in the 52 HC group. The proportion of ANR was significantly higher in the FEP group than in the HC group, with a ***χ***^***2***^ value of 6.247 (*p* = 0.012). Table 2 shows no significant differences were observed between the ANR and NNR groups regarding age, sex, education level, or BMI. In terms of clinical symptoms, the ANR group displayed significantly more severe negative symptoms (mean: 26.14 vs. 17.30, *p* = 0.001) and higher total scores on the PANSS (95.14 vs. 83.90, *p* = 0.014). Cognitive assessments indicated that the ANR group performed significantly worse on the Trail Making Test A (51.71 vs. 38.40, *p* = 0.010) and the BVMT-R (17.36 vs. 22.50, *p* = 0.041). Other cognitive measures, including BACS symbol coding, HVLT-R, WMS-3 spatial span, NAB mazes, Category Fluency, and CPT-IP, did not show significant differences between the two groups.

**Table 2:** Demographic, clinical and cognitive characteristics and comparisons between FEP patients with ANR and NNR groups.

Variables	ANR		NNR		Comparisons	
					*t**/χ***^***2***^	*p*
Cases [n, %]	14		40		–	–
Age (years) [mean, SD.]	24.07	9.466	24.93	8.438	−0.316	0.753
Male [n, %]	7	50.0 %	20	50.0 %	0	1
Female [n, %]	7	50.0 %	20	50.0 %		
Education (years) [mean, SD.]	11.50	3.858	12.10	3.053	−0.590	0.558
Father Education (years) [mean, SD.]	12.38	3.042	9.47	3.909	2.438	0.018
Mother Education (years) [mean, SD.]	10.31	3.568	8.82	4.203	1.145	0.258
Height (meter) [mean, SD.]	1.69	0.071	1.68	0.075	0.566	0.574
Weight (kg) [mean, SD.]	61.46	11.236	58.76	9.718	0.861	0.393
BMI [mean, SD.]	21.55	3.838	20.93	3.105	0.598	0.552
Positive symptoms [mean, SD.]	24.43	4.831	22.43	5.674	1.178	0.244
Negative symptoms [mean, SD.]	26.14	8.236	17.30	7.783	3.605	0.001
General symptoms [mean, SD.]	44.57	6.284	44.18	7.071	0.185	0.854
PANSS total score [mean, SD.]	95.14	14.628	83.90	14.140	2.538	0.014
Trail Making A	51.71	16.504	38.40	15.996	2.659	0.010
BACS symbol coding	42.71	7.043	47.95	12.426	−1.489	0.143
HVLT-R	16.93	7.839	19.65	5.890	−1.362	0.179
WMS-3 spatial span	13.29	2.614	14.60	2.854	−1.514	0.136
NAB mazes	10.07	5.636	13.08	6.788	−1.484	0.144
BVMT-R	17.36	8.261	22.50	7.799	−2.092	0.041
Category Fluency	15.64	5.624	17.35	5.162	−1.041	0.303
CPT-IP	1.94	1.038	2.09	0.741	−0.589	0.558

Note: BMI: Body Mass Index; SD: standard deviation; BACS, Brief Assessment of Cognition in Schizophrenia symbol coding; BVMT-R, Brief Visuospatial Memory Test–Revised; CPT-IP, Continuous Performance Test–Identical Pairs; HVLT-R, Hopkins Verbal Learning Test–Revised; NAB, Neuropsychological Assessment Battery mazes; WMS-3, Wechsler Memory Scale–Third Edition spatial span.

**Fig. 1:**
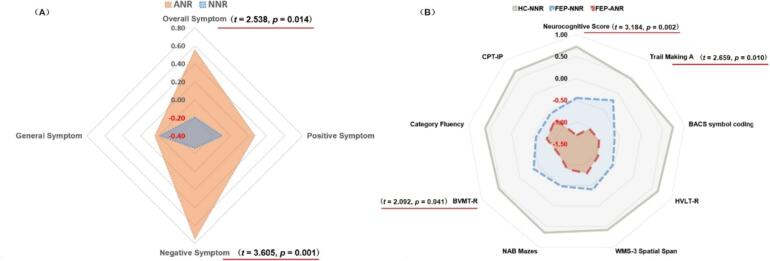
Radar chart comparing clinical and cognitive functioning among HC, ANR, and NNR groups. Note: *Z*-scores were calculated for various clinical and cognitive measures, including overall symptoms, positive symptoms, negative symptoms, and performance on cognitive tests. Significant differences among the groups are indicated, highlighting the deficits observed in the ANR group. Variables were Z-score transformed based on the full sample. The Z-score for Trail Making A was inversely processed and given a negative sign. BACS: Brief Assessment of Cognition in Schizophrenia symbol coding; BVMT-R: Brief Visuospatial Memory Test–Revised; CPT-IP: Continuous Performance Test–Identical Pairs; HVLT-R: Hopkins Verbal Learning Test–Revised; NAB: Neuropsychological Assessment Battery mazes; WMS-3: Wechsler Memory Scale–Third Edition spatial span. All *p*-values are reported without correction. For multiple comparisons, corrected thresholds were calculated as 0.05/3 = 0.017 for clinical symptom dimensions and 0.05/8 = 0.006 for cognitive tests.

To facilitate a clearer comparison of the differences among various clinical and cognitive variables, *Z*-scores were calculated and presented in a radar chart (Fig. 1). The results indicate that the ANR group exhibited significantly higher overall symptoms, negative symptoms and lower overall neurocognitive scores. Additionally, cognitive performance in the ANR group was notably poorer on the Trail Making Test A (*p* = 0.010) and BVMT-R (*p* = 0.041) the NNR group. For the clinical symptom dimensions, as there are three dimensions, the corrected *p*-value threshold is 0.05/3 = 0.017. The significant difference in negative symptoms remains valid after correction. For the cognitive tests, applying a corrected threshold of 0.05/8 = 0.006, no single cognitive test remains significant after correction. However, the composite neurocognitive score, which integrates all cognitive tests, still demonstrates a significant difference.

### 3.3 Correlation analysis

**Fig. 2:**
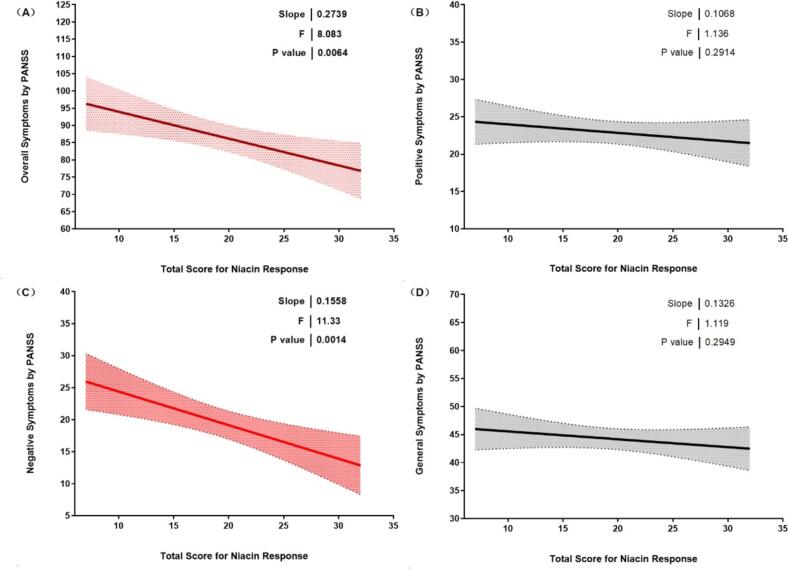
Correlation between symptoms and the degree of niacin skin response. Panel A displays the relationship for overall symptoms, while Panels B, C, and D focus on positive, negative, and general symptoms respectively. Statistical parameters including slope, *F*-value, and *p*-value are indicated for each panel.

Fig. 2 illustrates the correlation between symptom severity and the degree of niacin skin response. Panel A shows a significant positive correlation between overall symptoms, as measured by the PANSS, and total scores for niacin response (slope = 0.274, *F* = 8.083, *p* = 0.0064). Panel B presents the relationship between positive symptoms and niacin response, revealing no significant correlation. Conversely, Panel C indicates a significant positive correlation between negative symptoms and niacin response (slope = 0.156, *F* = 11.33, *p* = 0.0014). Finally, Panel D shows no significant correlation for general symptoms.

**Fig. 3:**
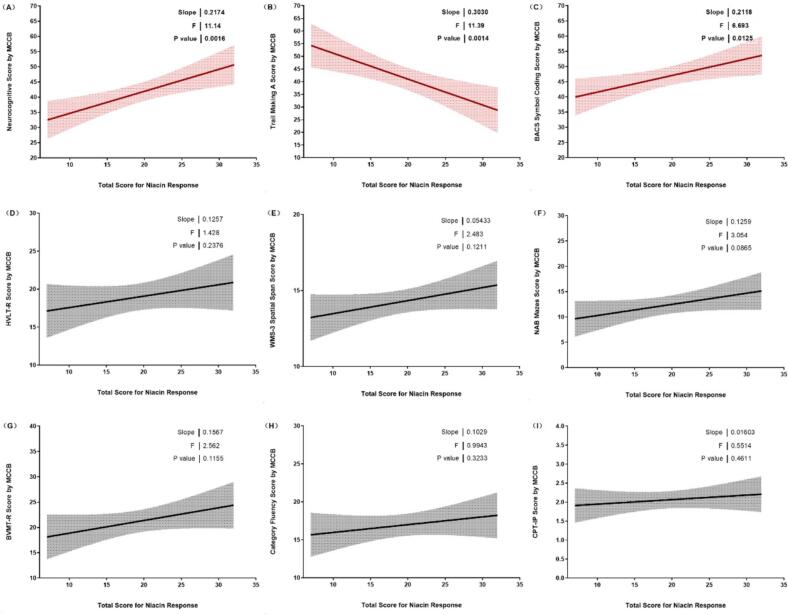
Correlation between cognitive performance and the degree of niacin skin response. Panels A, B, and C illustrate significant correlations for overall cognitive scores, Trail Making A, and BACS symbol coding respectively. Panels D through H display correlations for additional cognitive measures, with statistical parameters including slope, *F*-value, and *p*-value indicated for each panel. BACS: Brief Assessment of Cognition in Schizophrenia symbol coding; BVMT-R: Brief Visuospatial Memory Test–Revised; CPT-IP: Continuous Performance Test–Identical Pairs; HVLT-R: Hopkins Verbal Learning Test–Revised; NAB: Neuropsychological Assessment Battery mazes; WMS-3: Wechsler Memory Scale–Third Edition spatial span.

Fig. 3 depicts the correlation between cognitive performance and the degree of niacin skin response. Panel A shows a significant positive correlation between overall cognitive scores and total scores for niacin response (slope = 0.217, *F* = 11.14, *p* = 0.0016), indicating that higher niacin response is associated with better cognitive performance. Panel B presents the relationship for the Trail Making A, revealing a significant correlation (slope = 0.303, *F* = 11.39, *p* = 0.0014). In Panel C, the correlation for the BACS symbol coding is also significant (slope = 0.212, *F* = 6.69, *p* = 0.0125), further supporting the link between cognitive functioning and niacin response. Panels D through H illustrate various other cognitive measures, but none show significant correlations with niacin response.

Table 3 presents the correlations between clinical symptoms (positive, negative, and general) and cognitive performance across various domains. Among these results, only negative symptoms were significantly correlated with performance on the Trail Making A test (*r* = 0.366, *p* = 0.007), indicating a specific relationship between greater negative symptom severity and poorer performance on this task.

**Table 3:** Correlations between clinical symptoms and cognitive performance in FEP patients.

Variables	Positive symptoms		Negative symptoms		General symptoms	
	*r*	*p*	*r*	*p*	*r*	*p*
Trail Making A	0.250	0.069	0.366	0.007	0.115	0.408
BACS symbol coding	−0.062	0.655	−0.182	0.188	0.049	0.726
HVLT-R	−0.157	0.256	−0.243	0.077	−0.022	0.873
WMS-3 spatial span	−0.249	0.069	−0.262	0.055	−0.077	0.579
NAB mazes	−0.171	0.217	−0.146	0.293	0.080	0.565
BVMT-R	−0.113	0.416	−0.234	0.089	−0.023	0.872
Category Fluency	−0.053	0.705	−0.264	0.053	−0.042	0.762
CPT-IP	−0.206	0.135	−0.141	0.309	−0.026	0.854

Note: BACS, Brief Assessment of Cognition in Schizophrenia symbol coding; BVMT-R, Brief Visuospatial Memory Test–Revised; CPT-IP, Continuous Performance Test–Identical Pairs; HVLT-R, Hopkins Verbal Learning Test–Revised; NAB, Neuropsychological Assessment Battery mazes; WMS-3, Wechsler Memory Scale–Third Edition spatial span.

## 4 Discussion

This study has several strengths, including its focus on FEP patients who are drug-naive, the comprehensive and standardized cognitive assessments conducted, the use of standardized niacin skin flushing testing, and the inclusion of matched HCs. To the best of our knowledge, this research is the first investigation of the cognitive function characteristics of the ANR subtype specifically in a drug-naive FEP population. The key findings of this study indicate that patients with the ANR subtype of FEP exhibit more pronounced cognitive impairments compared to those in the NNR group. Notably, significant differences were observed in overall neurocognitive scores, as well as in specific tasks such as the Trail Making Test A and the BVMT-R. Additionally, ANR subtype patients presented with more severe clinical symptoms, particularly negative symptoms. These results suggest that the ANR subtype may represent a distinct group of patients within FEP characterized by core features of negative symptoms, cognitive deficits, and functional decline. This may point to a biological subtype associated with pathogenic mechanisms related to oxidative stress, phospholipid metabolism abnormalities, and inflammatory imbalance. (Ansarey, 2021; Xu et al., 2019).

The finding that 25.9 % of FEP patients were classified as having the ANR subtype highlights its importance in early psychosis. This proportion is slightly lower than the 31 % reported by Yao et al. (Yao et al., 2016), who used laser Doppler methods to investigate the distribution of ANR in independent samples from Pittsburgh and Portland. Their results indicated that the ANR proportion was 31 % in the Pittsburgh sample and 32 % in the Portland sample, suggesting that this subtype comprises approximately one-third of the patient population. While Yao et al. focused on stabilized schizophrenia patients, our research specifically involved drug-naive FEP individuals. It is essential to recognize that not all FEP patients progress to schizophrenia, which may explain the lower prevalence of the ANR subtype in our cohort. Moreover, the different methods employed for evaluating the niacin skin response might also have contributed to the observed difference in the prevalence of the ANR subtype between the two samples. In our study, we used visual assessment, whereas the study by Yao et al. utilized the Doppler method. This finding suggests that the ANR subtype may manifest more prominently in patients with more severe and advanced stages of psychotic disorders.

The clinical manifestations of the ANR subtype identified in this study, characterized by prominent negative symptoms and cognitive impairments, raise important considerations regarding their underlying causes and clinical implications. Negative symptoms, such as affective flattening and social withdrawal, may reflect a more profound neurobiological dysfunction in this subtype, potentially linked to alterations in neurotransmitter systems or neuroinflammatory processes (Galderisi et al., 2015; Galderisi et al., 2018; Goldsmith and Rapaport, 2020). Cognitive deficits, which are often associated with functional impairment, may further exacerbate the challenges faced by patients in their daily lives, impacting their social interactions and overall quality of life (Vita et al., 2022). The presence of these core features in the ANR subtype suggests that early identification and targeted interventions may be crucial for improving outcomes. Clinicians may need to adopt a more nuanced approach when assessing and treating patients with the ANR subtype, emphasizing the management of negative symptoms and cognitive rehabilitation. This could involve integrating psychosocial interventions and pharmacological strategies tailored to address these specific challenges. Recognizing the distinctive clinical profile of the ANR subtype could ultimately enhance personalized treatment plans and improve the prognosis for patients experiencing early psychosis.

The significant cognitive impairments observed in the ANR subtype, particularly in the Trail Making A and the BVMT-R, warrant further discussion. The Trail Making A primarily assesses processing speed(Miner and Ferraro, 1998), mental flexibility, and visual attention, all of which are crucial for effective functioning in daily life. Impairments in this domain may reflect underlying deficits in executive function and information processing that are commonly associated with the neurobiological abnormalities observed in the ANR subtype. Similarly, the BVMT-R evaluates visuospatial memory, which is essential for tasks that require the integration of visual and spatial information. Deficits in this area can significantly impact a patient's ability to navigate their environment and engage in activities that require memory recall and spatial awareness. The pronounced impairments in these specific cognitive domains in the ANR subtype may indicate that individuals with this classification experience more severe disruptions in the neural circuits responsible for these functions.

Our previous research extensively assessed cognitive function in CHR individuals (Cui et al., 2020; Zhang et al., 2023b; Zhang et al., 2024d), identifying visuospatial learning performance as a key predictor of psychosis development (Cui et al., 2020; Zhang et al., 2023b). We found that CHR individuals who later converted to psychosis consistently exhibited poorer BVMT-R performance compared to non-converting CHR individuals (Zhang et al., 2022a), highlighting the significance of visuospatial learning deficits in psychosis progression. Following this, our team conducted a proof-of-concept, randomized, sham-controlled clinical trial in which CHR patients underwent targeted transcranial magnetic stimulation (TMS) aimed at enhancing the network between the left inferior parietal lobule and the left hippocampus—an area critical for episodic memory retrieval and spatial navigation (Tang et al., 2023). This accelerated TMS protocol, consisting of 10 sessions of 20 Hz treatments over two days, demonstrated significant improvements in BVMT-R performance exclusively following active TMS, not sham TMS (Wang et al., 2014). These findings suggest that the left parieto-hippocampal TMS protocol can selectively enhance visuospatial learning performance, offering a potential therapeutic avenue for FEP patients with the ANR subtype.

Moreover, there is potential for developing novel treatments for the ANR subtype through nutritional interventions, although the current evidence base is limited and further research is needed. Regarding polyunsaturated fatty acid (PUFA), early studies indicated that they might reduce the risk of psychosis onset in individuals at clinical high risk (CHR) (Amminger et al., 2010), yet subsequent research yielded negative results (McGorry et al., 2017), suggesting that the effectiveness of PUFA interventions may not be consistent across all CHR individuals (Amminger et al., 2020). However, identifying patients with abnormal fatty acid metabolism seems to enhance the outcomes of PUFA interventions. For patients with the ANR phenotype, PUFA supplementation may be a beneficial approach to help mitigate disease risk. In addition, sulforaphane, known for its potent anti-inflammatory and antioxidant properties, has shown promise in the treatment of psychotic disorders and autism (Singh et al., 2014; Singh and Zimmerman, 2016). Some studies have reported its therapeutic effects, and ongoing clinical trials, such as those by Professor Zhao's team for first-episode schizophrenia (Xiao et al., 2021) and our team's randomized controlled trial for risk reduction in CHR individuals (Li et al., 2021), are further exploring its potential.

This study has several limitations that should be acknowledged. First, the sample size, while sufficient for preliminary findings, may limit the generalizability of the results. A larger and more diverse cohort would provide more robust insights into the cognitive characteristics of the ANR subtype across different populations. Second, the cross-sectional design of the study restricts the ability to draw causal inferences about the relationship between ANR status and cognitive function. Longitudinal studies would be beneficial to track cognitive changes over time and their potential impact on clinical outcomes. Additionally, while we utilized standardized assessments, the reliance on specific cognitive tests may not capture the full spectrum of cognitive impairments experienced by patients. Future research should consider a broader range of cognitive assessments such as social cognition (Zhang et al., 2018; Zhang et al., 2022b; Zhang et al., 2016b) to provide a more comprehensive understanding of cognitive dysfunction in the ANR subtype. Finally, the observational nature of the study limits the ability to control for all potential confounding variables, such as environmental factors or comorbid conditions, which may influence cognitive performance and symptom expression.

## 5 Conclusion

This study highlights the distinctive cognitive impairments associated with the ANR subtype of FEP, particularly in processing speed and visuospatial memory. The findings underscore the clinical significance of identifying this subtype, as it is characterized by more pronounced negative symptoms and cognitive deficits. By recognizing the unique profile of the ANR subtype, clinicians can develop targeted interventions aimed at addressing these specific challenges, potentially improving outcomes for patients. Further research is needed to explore the underlying mechanisms and to evaluate the effectiveness of tailored therapeutic strategies for individuals with the ANR subtype (Gao et al., 2024; Li et al., 2021), ultimately enhancing our understanding and management of early psychosis (Zhang et al., 2024e).

## CRediT authorship contribution statement

**MingLiang Ju:** Writing – review & editing, Writing – original draft, Methodology, Investigation, Conceptualization. **Bin Long:** Writing – review & editing, Writing – original draft, Conceptualization. **YanYan Wei:** Writing – review & editing, Methodology, Investigation. **XiaoChen Tang:** Writing – review & editing, Formal analysis, Data curation. **LiHua Xu:** Writing – review & editing, Methodology, Investigation. **RanPiao Gan:** Writing – review & editing, Methodology, Investigation. **HuiRu Cui:** Writing – review & editing, Methodology, Investigation. **YingYing Tang:** Writing – review & editing, Formal analysis, Data curation. **ZhengHui Yi:** Writing – review & editing, Resources, Methodology. **HaiChun Liu:** Writing – review & editing, Formal analysis, Data curation. **ZiXuan Wang:** Writing – review & editing, Investigation, Data curation. **Tao Chen:** Writing – review & editing, Formal analysis, Data curation. **Jin Gao:** Writing – review & editing, Methodology, Data curation. **Qiang Hu:** Writing – review & editing, Investigation, Formal analysis. **LingYun Zeng:** Writing – review & editing, Methodology, Investigation. **ChunBo Li:** Writing – review & editing, Visualization, Validation, Supervision, Resources. **JiJun Wang:** Writing – review & editing, Visualization, Validation, Supervision, Resources, Conceptualization. **HuanZhong Liu:** Writing – review & editing, Writing – original draft, Conceptualization. **TianHong Zhang:** Writing – review & editing, Writing – original draft, Visualization, Validation, Supervision, Funding acquisition, Formal analysis, Data curation, Conceptualization.

## Funding

This study was supported by the Ministry of Science and Technology of China, 10.13039/501100012166National Key Research and Development Program of China (2023YFC2506800), 10.13039/501100001809National Natural Science Foundation of China (82171544, 82371505, 82151314, 82101623), STI 2030-Major Projects (2022ZD0208500), Shenzhen Science and Technology Plan Project (JCYJ20220530165009020), Shenzhen Medical and Health Three Project (SZSM202011014), and Qingdao Science and Technology Benefit People Program (22–3-7-smjk-19-nsh).

## Declaration of competing interest

The authors report no biomedical financial interests or potential conflicts of interest.
